# The Response of the Gut Microbiota to Dietary Changes in the First Two Years of Life

**DOI:** 10.3389/fphar.2020.00334

**Published:** 2020-03-17

**Authors:** Yichen Li, Howard S. Faden, Lixin Zhu

**Affiliations:** ^1^Department of Colorectal Surgery, Guangdong Provincial Key Laboratory of Colorectal and Pelvic Floor Diseases, Guangdong Institute of Gastroenterology, the Sixth Affiliated Hospital, Sun Yat-sen University, Guangzhou, China; ^2^Department of Pediatrics, Division of Infectious Diseases, Jacobs School of Medicine and Biological Sciences, University at Buffalo, Buffalo, NY, United States; ^3^Genome, Environment and Microbiome Community of Excellence, State University of New York at Buffalo, Buffalo, NY, United States

**Keywords:** short chain fatty acids, butyric acid, acetic acid, propionic acid, bile acids, primary bile acids, secondary bile acids

## Abstract

The infant gut microbiota undergoes significant changes in the first two years of life in response to changes in the diet. The discontinuation of the milk-based diet of the first year and the introduction of solid foods in the second year of life results in a decline in *bifidobacterium*, a shift from infant strains of *bifidobacterium* to adult strains which preferentially metabolize oligosaccharides derived from plants rather than from milk, a surge in short chain fatty acids such as acetic, propionic and butyric acid from newly acquired commensal *clostridium*, and the transformation of primary bile acids into secondary bile acids by a limited number of newly acquired and highly specialized *Clostridium* spp. By 3 years of age, diet and gut microbiota closely resemble those of adults. Gut bacteria required for the production of SCFAs and secondary BAs are potential targets for the intervention of microbiome-related diseases.

## Introduction 

In the first year of life, human milk provides a large quantity and a broad array of complex oligosaccharides (HMO). While humans are unable to digest these complex oligosaccharides, strains of bifidobacterium commonly found in infants readily metabolize them (Milani et al., 2017; Turroni et al., 2018a). The large array of HMO is essential for the growth and survival of bifidobacterium. The cessation of human milk-derived oligosaccharides and the introduction of plant-derived oligosaccharides in the first 2 years of life forces a shift in the gut microbiome away from bifidobacterium and towards organisms belonging to the class of Clostridium (Browne et al., 2016; Guittar et al., 2019). The various organisms belonging to the Clostridium class are very specialized; they produce short chain fatty acids (SCFA) such as acetic, propionic, and butyric and transform primary bile acids (PBA) into secondary bile acid (SBA). The effects of SCFA and SBA on the host are both local and systemic. Short chain fatty acids regulate sodium and fluid absorption in the gut and maintain intestinal motility and the integrity of the mucosal epithelium as well serve anti-inflammatory mediators (Scheppach, 1994; Kelly et al., 2015; Zhu et al., 2015; Riviere et al., 2016; Hiippala et al., 2018).The aims of this review are to characterize the changes in the gut microbiota and their metabolic products in response to dietary changes and to highlight the importance of the gut microbiota and their products on overall health.

### Comparison of the Nutrient Composition of Human Milk and Cow Milk Formula

A normal diet in the first year of life consists human milk or cow milk-based infant formula. Both milk preparations contain fats, carbohydrates and proteins, although, the amounts and types of fats, carbohydrates, and proteins vary. For example, human milk contains 100 to 1,000 fold more oligosaccharides than cow milk and more than 250 types of fucosylated, nonfucosylated, and sialylated oligosaccharides. Fucosylated oligosaccharides predominate in human milk at 60%–80% and sialylated oligosaccharides comprise only 10%–15% (Ninonuevo et al., 2006). In contrast, fucosylated oligosaccharides comprise less than 1% of cow milk oligosaccharides while sialyloligosaccharides comprise 85% (Boehm, 2003; Milani et al., 2017; Bell et al., 2018). Manufacturers of infant formula often add galactooligosaccharides and/or fructooligosaccharides to cow milk formula to more closely mimic human milk. When infants are weaned from either human milk or cow milk formula, there is a sudden loss of oligosaccharides and a resultant decline of bifidobacterium populations with a few exceptions discussed below (Turroni et al., 2018a).

### Processing Dietary Complex Oligosaccharides by Bifidobacterium

During the first year of life, *bifidobacterium* colonize 100% of infants and comprise as much as 90% of the gut microbiota in human milk-fed infants; less *bifidobacterium* and more bacteroides are typical of formula-fed infants (Yatsunenko et al., 2012; Underwood et al., 2015; Nagpal et al., 2017). *Bifidobacterium* dominate the infant gut microbiota because they are capable of digesting complex milk oligosaccharides. Among the more than 50 types of *bifidobacterium*, *Bifidobacterium breve*, *B. bifidum*, and *B. infantis* are most common in the infant gut. These strains possess enzymes which are able to ferment the various milk oligosaccharides (Katayama, 2016). *B. breve*, however, is unique and persists in large numbers despite the discontinuation of human milk or cow milk formula because it has the capability to utilize sialylated oligosaccharides found in plants and cow milk (Turroni et al., 2018a). Avershin et al. (2016) suggest that *B. breve* is a critically important evolutionary link between the infant and adult gut microbiome.

### The Effect of Dietary Changes on the Gut Microbiota in the First 2 Years of Life

The cessation of milk oligosaccharides and the addition of solid foods results in a 10 to 100 percent decline in the abundance of *bifidobacterium* and an increase in the numbers of commensal *clostridium* and *bacteroides* (Bergstrom et al., 2014; Backhed et al., 2015; Davis et al., 2016; Khonsari et al., 2016). Work done by Bakhed and associates (Backhed et al., 2015) suggests that the cessation of breast-feeding is the major contributor to the functional shift in the gut microbiome. Despite a reduction in *bifidobacterium* numbers in the adult gut, *bifidobacterium* continues to play a substantial role in the metabolism of dietary complex oligosaccharides and host-derived oligosaccharides, such as colonic mucin (Hao et al., 2019). Strains of *bifidobacterium* commonly associated with the adult gut such as *B. longum*, *B. adolescentis*, and *B. catenulatum* characteristically ferment oligosaccharides derived from fruit, vegetables, and grains (De Leoz et al., 2015; Arboleya et al., 2016; Turroni et al., 2018a; Turroni et al., 2018b). *B. longum*, in particular, has an unusually high genomic capacity to utilize plant-derived glycans, especially non-fucosylated/non-sialylated oligosaccharides (Hiippala et al., 2018). *B. bifidum*, a strain commonly found in infants, possesses the ability to utilize host-derived glycans found in intestinal mucins which suggests a co-evolutionary relationship between the humans and *B. bifidum*. (Hao et al., 2019). The diversity of carbohydrate utilization exhibited by *bifidobacterium* insures the survival of specific species in the human gut microbiota (Turroni et al., 2018a).

A majority of infants will continue to consume large amounts of cow milk throughout their childhood and into their adulthood. Much of the sialyloligosaccharides in cow milk are linked to glycoproteins and glycolipids (Lis-Kuberka and Orczyk-Pawilowicz, 2019). Sialylated compounds are incorporated in the nervous system and enhance the neurodevelopment of infants. They may also function as decoys to inhibit sialic acid-dependent enteric pathogen such as *E. coli*, rotavirus, cholera toxin, *Salmonella* spp., and *Shigella* spp. from attaching to intestinal epithelium and causing disease (Lis-Kuberka and Orczyk-Pawilowicz, 2019). While cow milk oligosaccharides have been shown to enhance growth in animal models, their effects on growth, development and health of children awaits further investigation (Charbonneau et al., 2016).

### Specialized Commensal *Clostridium* and the Production of Short Chain Fatty Acids

As stated earlier, the cessation of human milk and the introduction of solid foods initiates a dramatic shift in the bacterial population of the gut. Specifically, *clostridium* and *bacteroides* become the major commensal gut organisms during the second year of life (Lopetuso et al., 2013; Bergstrom et al., 2014). In fact, the abundance of *clostridia* and *bacteroides* doubles in the second year of life resulting in a sustained surge of short chain fatty acids (SCFA) (Koenig et al., 2011; Laforest-Lapointe and Arrieta, 2017). Of these two groups of organisms, *clostridia* are the largest contributor to the production of SCFA acetic, propionic, and butyric. The order Clostridiales is an extremely large group of organisms which includes the genera *Anaerostipes, Clostridium, Coprococcus, Dorea, Eubacterium, Faecalibacterium, Roseburia, Ruminococcus, Peptococcus*, and *Peptostreptococcus* which are involved in SCFA production (Collins et al., 1994).

### The Importance of Three SCFA in Maintaining Health

SCFAs consist of five or less carbons. They are products of carbohydrate fermentation and, to a lesser degree, protein metabolism by anaerobic bacteria. In mammals, formic acid, acetic acid, propionic acid, butyric acid, isobutyric acid, valeric acid, and isovaleric acid comprise the list of SCFAs. While simple sugars are mostly absorbed or digested by brush border enzymes in the small intestine, the more complex carbohydrates cannot be absorbed or digested by humans; they are, however, metabolized by the aforementioned members of the clostridium class in the colon (Browne et al., 2016; Guittar et al., 2019). The highest concentrations of SCFA are found in the cecum and the lowest in the more distal segments of the colon (Cummings et al., 1987). The acidity in different segments of the colon is directly proportional to SCFA levels.

**Figure 1:**
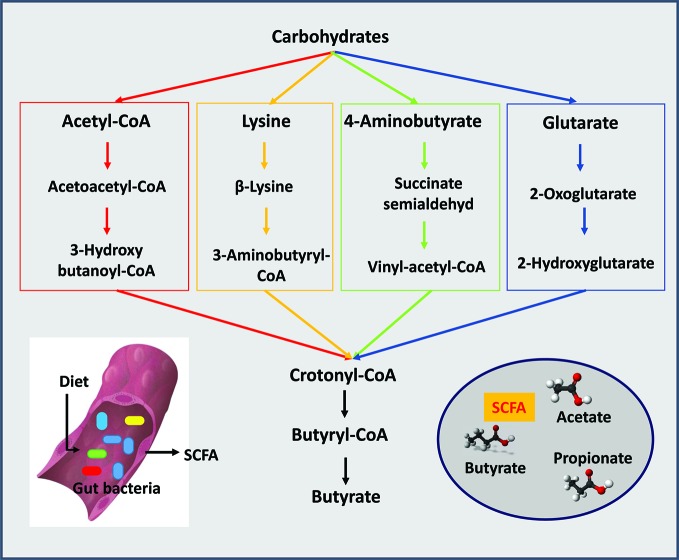
Short chain fatty acids (SCFAs) are produced by gut bacteria. The three major SCFAs are acetate, propionate, and butyrate. Four pathways are known for the production of butyrate from carbohydrates: the acetyl-coenzyme A pathway is the most prevalent followed by the lysine pathway, 4-aminobutyrate and glutarate-based pathways.

Acetic acid is the most abundant SCFA in the gut, exceeding propionic and butyric acid levels by two fold (Cummings, 1981; Koenig et al., 2011). Most anaerobes in the colon produce acetic acid. *Bacteroides* spp. produce acetic and propionic acids as does *Akkermansia muciniphila* (Sghir et al., 2000; Koenig et al., 2011). *Clostridium* organisms are somewhat unique because they produce all three major SCFA (Cummings and Macfarlane, 1991; Lopetuso et al., 2013). Among the major SCFA, butyric acid is the most essential for the maintenance of a healthy gut. Several synthesis pathways exist for the production of butyric acid from carbohydrates (**Figure 1**). The acetyl-coenzyme A pathway is the most prevalent followed by the lysine pathway; 4-aminobutyrate and glutarate-based pathways are least abundant (Vital et al., 2014). In general, organisms such *anaerostipes*, *clostridium*, *coprococcus*, *dorea*, *eubacterium*, *faecalibacterium*, *roseburia*, and *ruminococcus* all produce butyric acid; *eubacterium*, *roseburia*, and *faecalibacterium* are the most abundant producers (Louis and Flint, 2009; Hiippala et al., 2018). *Eubacterium* and *anaerostipes* interact with the *bifidobacterium* to enhance their butyric acid production capacity. *Bifidobacterium* participates in butyric acid production by generating acetic acid through fermentation of carbohydrates. Then, through a process called "cross-feeding" supply acetic acid to *eubacterium* and *anaerostipes* for butyric acid production (Turroni et al., 2018b). The "cross feeding" capabilities of infant strains, *B. bifidum* and *B. breve*, are taken over by adult strains of *bifidobacterium* and by the end of the second year of life adult patterns of SCFA production are achieved (Roy et al., 2006). Among all strains of *bifidobacterium*, *B. bifidum* has the unique capability to generate acetic acid *via* fermentation of intestinal mucins (Bunesova et al., 2018). *A. muciniphila*, a non *bifidobacterium* and the only *verrucomicrobium* found in the human intestine, is also capable of converting acetic to the butyric acid through fermentation of intestinal mucins (Derrien et al., 2004).

SCFAs perform many critical functions in humans (Zhu et al., 2015). In general, they stimulate colonic sodium and fluid absorption and they maintain acidic environment in the lumen of the colon. However, acetic acid, propionic acid, and butyric acid each possess specific physiologic functions (Roy et al., 2006). For example, acetic acid increases colonic blood flow and ileal motility and is anti-inflammatory (Scheppach, 1994; Riviere et al., 2016). Propionic acid is anti-inflammatory as well (Hiippala et al., 2018). Acetic and propionic acids are both absorbed into the circulation and affect tissues distant from the colon; in contrast, butyric acid conducts critical functions mostly within the gut. Butyric acid provides fuel for growth and differentiation of intestinal cells, stimulates mucin production and tightens cell junctions; each of these functions helps to increase the integrity of the colon epithelium (Hiippala et al., 2018). Butyric acid similarly augments oxygen consumption by colonic epithelial cells which in turn stimulates hypoxia-inducible factor (HIF) production to maintain the integrity of the barrier function of the gut (Kelly et al., 2015). Butyric acid aids the gut immune system and contributes to the prevention of inflammatory disorders such as colitis. Butyric acids’ anti-inflammatory activity may contribute to the prevention of colon cancer (Riviere et al., 2016). In order to accomplish the myriad of activities, butyric acid activates G-coupled-receptors and inhibits histone deacetylases (Koh et al., 2016). The contribution of butyric acid can be seen as early as 3–6 months of age with the over expression of butyrate synthesis enzymes in gut microbiota at a time when most butyric acid producing organism are rare (Gosalbes et al., 2019).

### The Role of *Clostridium* Species in the Transformation of Primary Bile Acid Into Secondary Bile Acids

**Figure 2:**
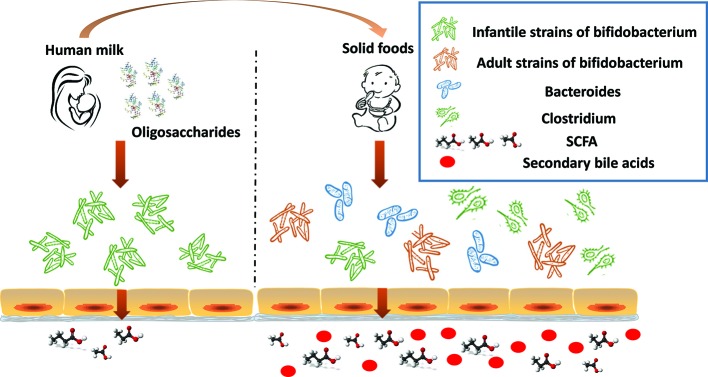
The infant gut undergoes significant changes in the first 2 years of life. In response to the dietary change (human milk oligosaccharides disappear), infant strains of bifidobacterium rapidly decline. As the proportion of dietary solid foods increase, adult strains of bifidobacterium proliferate, commensal clostridium, and bacteroides increase, as do bacteria capable of producing short chain fatty acid (SCFA), and secondary bile acids appear.

Additional changes to the gut microbiome in response to the introduction of solid foods in year two of life include the appearance of *Clostridium hiranonis, C. hylemonae, C. leptum, C. scindens*, and *C. sordellii*. These organisms along with *eubacterium* and *bacteroides* transform primary bile acids into secondary bile acids for the first time since birth (**Figure 2**) (Hylemon and Stellwag, 1976; Doerner et al., 1997; Kitahara et al., 2001). They possess dehydroxylases capable of removing hydroxyl groups from the α3, 7 and 12 carbon position in primary bile acids. Since the hydroxyl groups at the 7 carbon position is most susceptible to dehydroxylation, it is removed most often. Dehydroxylases responsible for removal of the 7 carbon hydroxyl group are regulated by a series of bile acid inducible gene operons (Doerner et al., 1997; Ridlon et al., 2016). Dehydroxylation increases the hydrophobicity of the bile which, in turn, increases membrane binding, and toxic and metabolic effects (Ridlon et al., 2006). The bacteria responsible for the formation of secondary bile acids are, however, resistant to the toxic effects of secondary bile acids; in contrast, pathogenic strains such as *Clostridium difficile* are susceptible, thus, reducing the risk of colonization and disease (Studer et al., 2016). The toxicity of secondary bile acids may extend beyond enteric pathogens such as *C. difficile* to the more common resident microbiota. Gram negative organisms, in particular, are more susceptible to secondary bile acids due to lipopolysaccharide (LPS) in cell wall than are Gram positive organisms which lack LPS. It seems reasonable to suggest that reducing the number of Gram negative bacteria reduces the dangers posed by endotoxin leaking through the gut wall. Kang et al. (2019) recently proposed that the production of tryptophan–derived antibiotics by 7α dehydroxylating commensal clostridium may enhance the toxicity of secondary bile acid toward *C. difficile*.

High levels of secondary bile acids in the gut and in the enterohepatic circulation may be oncogenic and lead to cancer in the colon and liver (Ridlon et al., 2016; Jiao et al., 2018). Fortunately, clostridium and bacteroides organisms are capable of decreasing the toxicity of secondary bile acids. These organisms possess hydroxysteroid dehydrogenases which can epimerize hydroxyls from α to the β position at the 7 carbon and thus render the bile acid less toxic (Ridlon et al., 2006; Ridlon et al., 2016). Future studies with hydroxysteroid dehydrogenases-containing probiotics may focus on therapeutic reduction of secondary bile acids through epimerization (Ridlon et al., 2006).

The effects of bile acids results from stimulation of receptors expressed in multiple tissues. Activation of signaling pathways involve the nuclear receptors Farnesoid X receptor (FXR) and G-coupled receptors TGR5 (Skelly et al., 2019). Secondary bile acids LCA and DCA are potent agonists for TGR5 (Maruyama et al., 2002) while the primary bile acid CDCA is the most potent agonist for FXR (Parks et al., 1999; Yu et al., 2002). Both receptors are highly expressed in hepatic and intestine tissues (Xu, 2011). Activation of these receptors helps regulate bile acid metabolism and inhibits inflammatory processes (Lee et al., 2006; Pols et al., 2011). Individually the FXR receptor is involved with lipid and glucose metabolism, hepatic regeneration, and intestinal bacterial growth (Ding et al., 2015). The TGR5 receptor affects body weight, glucose metabolism, modulates immune responses, and affects liver function (Pols et al., 2011).

### Potential Intervention Strategies for Microbiota-Related Diseases

Besides dietary changes, other environmental factors such as maternal microbiome (Mueller et al., 2015; Ferretti et al., 2018) are known to influence the gut microbiota and may cause dysbiosis in the gut of infants. For example, gestational diabetes mellitus can alter the microbiota of pregnant women and neonates at birth (Wang et al., 2018). As the microbiota may have profound impact on the health of the infants through microbial metabolites such as SCFAs and BAs, it is expected that dysbiosis may contribute to the pathogenesis of microbiota-related diseases and that microbial intervention may have some beneficial effects on these diseases. Several intervention strategies are being considered for microbiota-related diseases of infants. First, dietary changes may have profound effects on the gut microbial composition and thus influence disease pathogenesis in a way similar to prebiotics (Medina et al., 2018). Second, probiotic intervention may have beneficial effects on the microbiota and the host as well. Infant colic, a condition that severely impacts family quality of life, is correlated with altered abundance of *enterobacteria*, *bifidobacteria*, and *lactobacilli*. Several clinical trials report effectiveness of probiotics treatment for colic (Tintore et al., 2017). Third, effective interventions for various diseases with microbial products SCFAs (Gill et al., 2018) and BAs (Neuschwander-Tetri et al., 2015) have been reported with adults. Their effectiveness for pediatric patients awaits further study.

## Conclusions

Dietary changes in the first 2 years of life result in significant changes in the gut microbiota. These newly acquired microbes are critical for short chain fatty acid production and formation of secondary bile acids throughout life. These microbes are potential targets for the intervention of microbiota-related diseases.

## Author Contributions

Conceptualization: HF and LZ. Writing: YL, HF, and LZ.

## Funding

This work was partly supported by the National Natural Science Foundation of China (81770571), and funds from the University at Buffalo Community of Excellence in Genome, Environment and Microbiome (GEM).

## Conflict of Interest

The authors declare that the research was conducted in the absence of any commercial or financial relationships that could be construed as a potential conflict of interest.
